# The influence of prosthetic positioning and proximal femoral morphology on leg length discrepancy and early clinical outcomes of cementless total hip arthroplasty

**DOI:** 10.1186/s13018-023-03847-w

**Published:** 2023-06-05

**Authors:** Zhenchao Huang, Zian Zhang, Xinzhe Lu, Yikai Liu, Haining Zhang

**Affiliations:** Department of Joint Surgery, The Affiliated Hospital of Qingdao University, Qingdao, 266000 Shandong China

**Keywords:** Total hip arthroplasty, Leg length discrepancy, Canal flare index, Canal fill ratio, Center of rotation

## Abstract

**Background:**

Leg length discrepancy (LLD) is a common complication of total hip arthroplasty (THA). However, the relationship between femoral prosthesis filling, proximal femoral morphology, and acetabular prosthesis positioning with postoperative LLD and clinical outcomes is unclear. The aims of this study were to investigate the influence of canal flare index (CFI), canal fill ratio (CFR), center of rotation (COR), and femoral offset (FO) on (1) postoperative LLD; and (2) clinical outcomes in the two stem designs with different coating distribution.

**Methods:**

The study cohort included 161 patients who underwent primary cementless THA between January 2021 and March 2022 with either proximal coating or full coating stems. Multivariate logistic regression was used to assess the effect of CFI, CFR, COR, and FO on postoperative LLD, and linear regression to assess their effect on clinical outcomes.

**Results:**

No statistical difference was found in clinical outcomes or postoperative LLD between the two groups. High CFI (*p* = 0.014), low ΔVCOR (*p* = 0.012), and Gender (*p* = 0.028) were found independent risk factors for LLD one day postoperative. High CFI was also an independent risk factor for postoperative subjectively perceived LLD (*p* = 0.013). CFR at the level of 2 cm below the LT (*p* = 0.017) was an independent risk factor for Harris Hip Score.

**Conclusions:**

Proximal femoral morphology and acetabular prosthesis positioning but not femoral prosthesis filling affected the LLD. High CFI was an independent risk factor for postoperative LLD and subjectively perceived LLD, and low ΔVCOR was also an independent risk factor for postoperative LLD. Women were susceptible to postoperative LLD.

## Introduction

The use of cementless femoral stems for total hip arthroplasty (THA) is becoming the mainstream approach [1, 2], as it potentially preserves femoral bone stock with adequate primary stability as well [3]. Despite the occurrence of some specific complications such as prosthetic subsidence and stress shielding induced by oversizing [4], recent studies have shown excellent long-term survival rates of cementless femoral stems in THA [5, 6].

Leg length discrepancy (LLD) is a common complication of cementless THA due to inappropriate stem size option prone to human error, therefore achieving equalized postoperative leg length in primary THA remains challenging for orthopedic surgeons [7, 8]. LLD could lead to problems which impair patient's postoperative hip function and satisfaction rate [2, 9]. According to a study by O’Brien, gait disorders may develop if a 7–10 mm lengthening of the affected limb relative to the healthy limb was perceived [10]. Proper reconstruction of postoperative LLD is critical for clinical functional outcomes and patient satisfaction [11]. Leg length change after THA depends on the vertical protrusion distance of the cementless femoral stem relative to the proximal femur which is related to the size/design of the cementless femoral stem, proximal femoral morphology [12] and positioning of acetabular component [13], and may also be related to the patient's age, gender, and body mass index (BMI) [14–16]. The excessive vertical protrusion distance may lead to excessive leg length extension [17]. Canal fill ratio (CFR) [1] is a common criterion to describe the matching of the femoral stem to the femoral canal, while canal flare index (CFI) [12] for proximal femoral morphology. CFR and CFI can accurately describe prosthetic positioning which may result in inconsistent leg length [18, 19], and are wildly used in clinical practice. Different CFI and CFR can potentially cause different depths of penetration and later prosthetic subsidence [1, 19, 20], thus affecting the length of the affected limb. It has been shown that CFI was an independent risk factor for LLD [12]. Center of rotation (COR) and femoral offset (FO) are commonly used position parameters of the acetabular prosthesis [21]. It has been shown that the higher the COR of the hip was placed, the lower the length of the affected limb was correspondingly [13]. Inadequate reconstruction of the FO can result in reduced arm force of the abductor muscles and ultimately also lead to LLD and claudication [21, 22]. Understanding the effect of CFR, CFI [23] and acetabular component positioning [13] on leg length reconstruction can assist in optimizing prosthesis design and improve postoperative clinical outcomes. However, to the best of our knowledge, there is no comparative study conducted investigating the effect of CFR, CFI, COR, and FO on LLD between prostheses with different coatings/designs.

Literature has shown better radiographic outcomes and osseointegration in proximally coated compared to fully coated stems [24]. Yet it is unclear whether there are differences in clinical outcomes and LLD between different coated stems, and understanding the effect of stem design on LLD and clinical outcomes can help surgeons choose the proper prosthesis before surgery.

The aim of this study was to compare early clinical outcomes and postoperative LLD between two types of cementless femoral stems with different coating areas. And to identify the effect of CFI, CFR, and acetabular prosthesis positioning on 1) early clinical outcomes and 2) postoperative LLD (radiographic LLD and self-perception of LLD) by taking into account the confounding variables such as gender, age, BMI, and the type of femoral stem (proximally coated and fully coated).

## Materials and methods

### Patients

After institutional review board approval was obtained (QYFY WZLL 27467), 161 patients who underwent primary cementless THA between January 2021 and March 2022 at our university hospital were retrospectively reviewed in this study. Inclusion criteria were patients who underwent primary cementless THA for end stage osteoarthritis of hip, either with fully coated or proximally coated femoral stems. Patients who underwent bilateral THA, or lost to follow-up were excluded. Patients with a definite reduction in the contralateral joint space were also excluded.

All surgeries were performed by one experienced joint surgeon at the same center, using a modified Hardinge approach. Femoral stem type was selected by the joint surgeon based on the patient's individual condition. Based on preoperative anteroposterior radiographs of the pelvis, fully coated femoral stems were used for patients with a significantly wide medullary cavity and a thick femoral cortex, which was to improve osseointegration and reduce the risk of postoperative prosthesis loosening and subsidence. In addition, fully coated stems were also implanted in older or osteoporotic patients to improve osseointegration and long-term prosthesis survival rate. All patients received the same standard intraoperative and postoperative protocols. The mean follow-up period for patients was 17.66 ± 4.34 months (range 12 months to 26 months).

### Implants

**Fig. 1:**
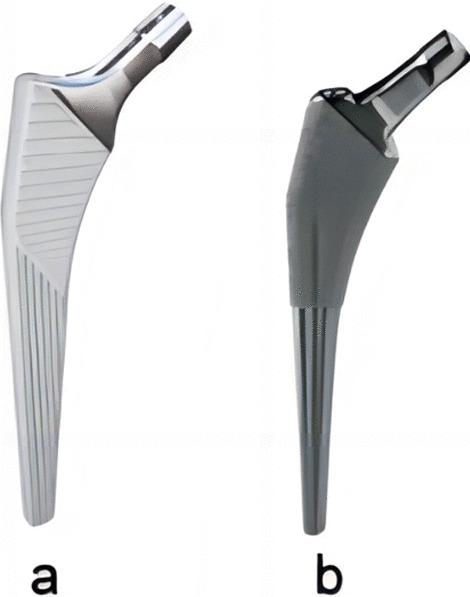
**a** Coronal profile of LCU® femoral stem. **b** Coronal profile of Taperloc® femoral stem

The LCU® stem (Waldemar Link GmbH & Co. KG, Barkhausenweg, Hamburg, Germany) is made of titanium alloy with a plasma-sprayed HA coating on the entire stem surface (Fig. 1a). The Taperloc® stem (Zimmer Biomet, Warsaw, Indiana, USA) is made of titanium alloy with a proximal plasma-sprayed PPS coating (Fig. 1b).

### Clinical analysis

During the follow-up, the Harris Hip Score (HHS) [25] and Forgotten Joint Score-12 (FJS-12) [26] were recorded at six months and one year after surgery, as well as patients’ subjective perception of inconsistent leg length one year after surgery. All assessments were performed by the same experienced joint surgeon who was not involved in the surgery. We contacted all 161 patients in the clinic or by telephone during the follow-up of more than 12 months, and no patients were missed.

### Radiographic analysis

**Fig. 2:**
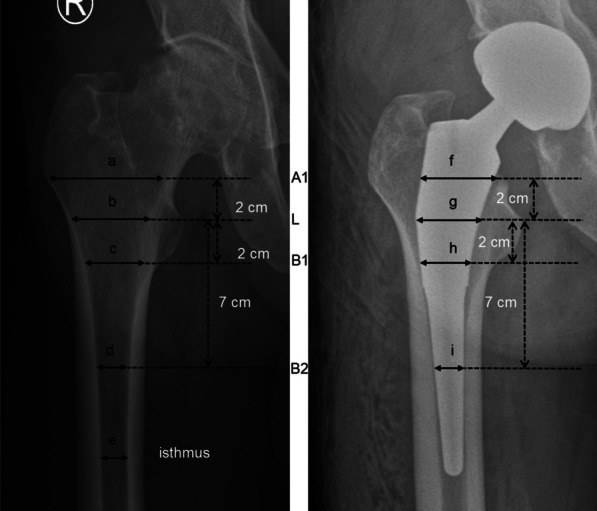
Measuring the CFI and CFR at 4 levels (A1, L, B1, B2). CFR (2 cm above the LT tip) = f/a, CFR (LT tip) = g/b, CFR (2 cm below the LT tip) = h/c, CFR (7 cm below the LT tip) = i/d. CFI = a/e

**Fig. 3:**
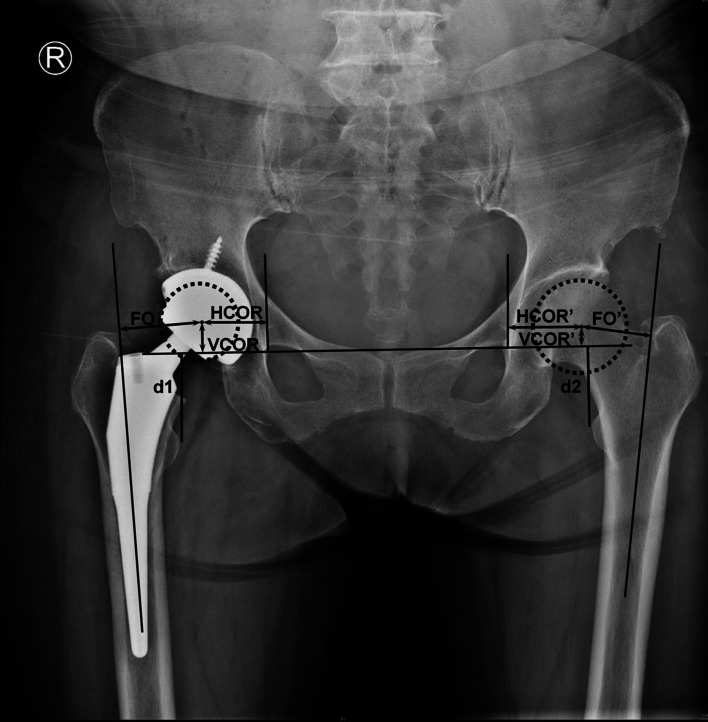
This figure is a schematic showing the method of calculating LLD. LLD is measured as the difference in the distance from the line joining the bottom edges of the two teardrops to the tip of each LT. Use the distance on the affected side (d1) minus the distance on opposite side (d2). HCOR was measured as the distance from the center of the femoral head to the vertical line passing through the inner edge of the ipsilateral teardrop. VCOR was measured as the distance from the center of the femoral head to the “inter-teardrop” line. FO was the perpendicular distance from the center of the femoral head to the longitudinal axis of the femur. Δ means the affected side distance minus the contralateral distance

Anteroposterior radiographs of the pelvis were performed preoperatively and day one postoperatively for radiographic measurements. All radiographic measurements were assessed by an independent observer who was not involved in the patients’ treatment. Four levels were defined [1]: A1, 2 cm above the lesser trochanter (LT) tip; L1, at the level of the LT tip; B1, 2 cm below the LT tip; B2, 7 cm below the LT tip. On radiographs day one postoperative, dividing the width of the stem by the corresponding width of the medullary canal at four levels (A1, L, B1, B2) as CFR (Fig. 2). On radiographs day one preoperative, dividing the width of the canal at A1 by the width of the canal isthmus as CFI (Fig. 2) [4, 18]. Radiographic LLD (Fig. 3) was defined as the difference between the distance from the tip of the LT to the line connecting the lowest points of the teardrops [27, 28]. Use the affected side distance (d1) minus the contralateral side distance (d2), take the affected side longer as the positive. COR and FO are both position parameters of acetabular prosthesis [21]. COR (Fig. 3) contains both horizontal (HCOR) and vertical center of rotation (VCOR). HCOR was defined as the distance from the center of the femoral head to the vertical line passing through the inner edge of the ipsilateral teardrop [29]. VCOR was defined as the distance from the center of the femoral head to the “inter-teardrop” line [30]. FO (Fig. 3) was defined as the perpendicular distance from the center of the femoral head to the longitudinal axis of the femur [30]. Δ means the affected side distance minus the contralateral distance.

### Statistical analysis

Continuous variables are expressed as mean ± standard deviation (SD), while categorical variables are expressed as number and percentage. All data were tested for normality using the Shapiroe–Wilk test. Independent samples t-test was used to compare normally distributed variables, while Mann–Whitney U test was used to compare non-normally distributed variables. Chi-square tests were used to compare categorical variables.

Multivariate linear regression was used to identify independent risk factors for HHS 6 months and 1 year postoperative. While multivariate logistic regression was used to identify independent risk factors for one day postoperative LLD and subjectively perceived LLD. For both of these regression analyses, exploratory univariate regression analysis was first performed to select possible risk factors associated with clinical outcomes or LLD among demographic parameters, radiographic parameters (CFI, CFR, COR and FO), and femoral prosthesis design. Variables with *p* < 0.2 were then included in a multivariate regression model to determine the independent effect of each factor. It has been shown that LLD > 10 mm adversely affects clinical outcomes [31]. Therefore, a binary output variable (0 for LLD < 10 mm; 1 for LLD > 10 mm) was defined. The level of significance was set at 0.05.

All statistical analyses were performed with IBM SPSS Statistics 26 (IBM Corp, Armonk, NY) software.

## Results

### Demographic characteristics

A total number of 161 patients were enrolled in our study, 99 patients received fully coated femoral stems and 62 patients received proximally coated stems. There was no significant difference in age, BMI, gender, and affected side between the two groups (Table 1).

**Table 1:** Demographic characteristics and postoperative clinical outcomes of patients (M ± SD)

	Fully coated stem group (*n* = 99)	Proximally coated stem group (*n* = 62)	*p* value
Age (year)	61.36 ± 9.81	59.89 ± 11.27	0.362
High (cm)	166.22 ± 7.94	167.42 ± 8.51	0.506
Weight (kg)	71.44 ± 11.06	71.02 ± 12.12	0.820
BMI (kg/m^2^)	25.81 ± 3.29	25.26 ± 3.30	0.306
*Gender (number, %)*			0.225
Male	61, 61.62%	44, 70.97%	
Female	38, 38.38%	18, 29.03%	
*Side (number, %)*			0.478
Left hip	52, 52.53%	29, 46.77%	
Right hip	47, 47.47%	33, 53.23%	
*Subjective LLD (number, %)*			0.140
Subjective LLD-positive	23, 23.23%	21, 33.87%	
Subjective LLD-negative	76, 76.77%	41, 66.13%	
HHS 6 months after surgery	84.27 ± 7.70	83.05 ± 7.28	0.282
HHS 1 year after surgery	93.39 ± 4.79	93.21 ± 4.87	0.840
FJS-12 6 months after surgery	63.59 ± 8.62	62.90 ± 8.19	0.665
FJS-12 1 year after surgery	79.25 ± 4.79	78.09 ± 4.74	0.100

*M ± SD* Mean ± Standard deviations, *BMI* Body mass index, *LLD* leg length discrepancy, *HHS* Harris Hip Score, *FJS-12* Forgotten Joint Score-12

### Radiographic LLD

**Fig. 4:**
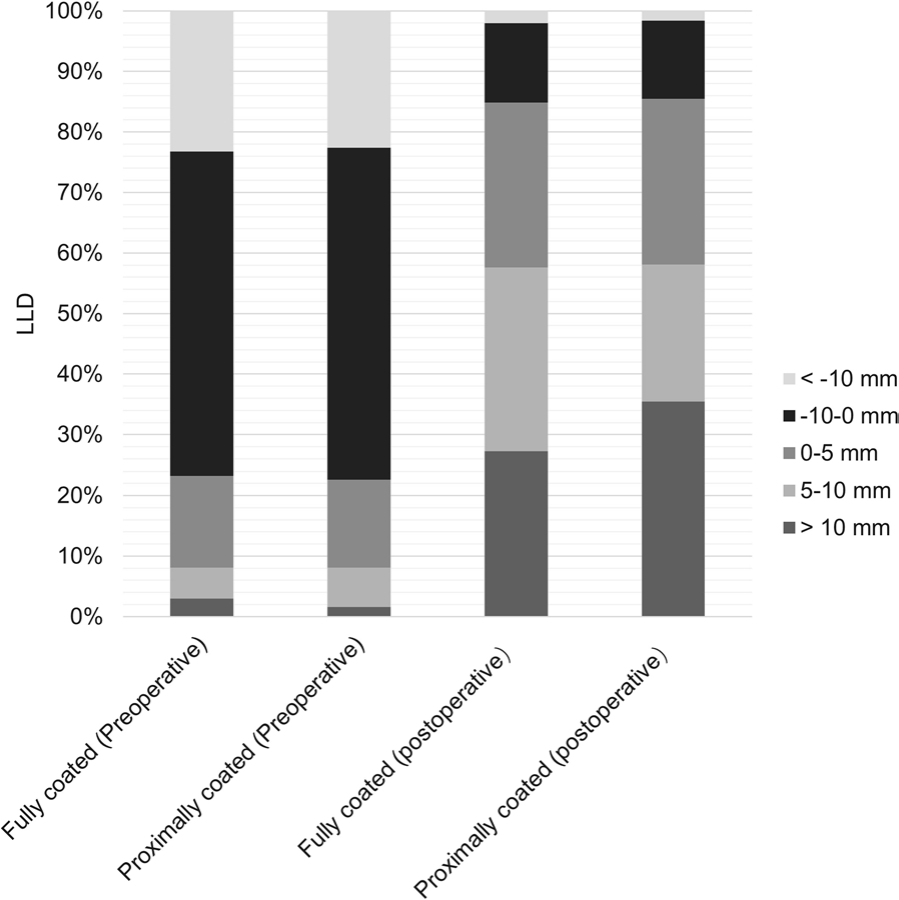
The bar chart shows the percentage of leg length difference before and after surgery. LLD was divided into 5 groups, which were LLD > 10 mm, 10 mm > LLD > 5 mm, 5 mm > LLD > 0 mm, 0 mm > LLD > − 10 mm and LLD < − 10 mm

Patients implanted with fully coated femoral stems had a significantly higher CFR at B2 compared to patients implanted with proximally coated stems (*p* < 0.001), with no significant difference in CFI or other levels of CFR (Table 2). There was also no significant difference in acetabular component positioning including ΔHCOR, ΔVCOR and ΔFO between the two groups. LLD did not differ between the two groups at either preoperative or one day postoperative (Table 2). Patients with preoperative LLD > 10 mm accounted for 3.03% in the fully coated group and 1.61% in the proximally coated group, respectively. Patients with postoperative LLD > 10 mm accounted for 27.27% in the fully coated group and 35.48% in the proximally coated group, respectively (Fig. 4). Postoperative LLD (fully coated, 5.99 ± 7.36 mm; proximally coated, 7.01 ± 7.50 mm) was significantly larger than preoperative LLD (fully coated, − 5.52 ± 8.96 mm; proximally coated, − 5.30 ± 7.70 mm) in both groups (*p* < 0.001) (Table 2).

**Table 2:** Comparison of radiographic parameters and results between two groups

	Fully coated stem group (*n* = 99)	Proximally coated stem group (*n* = 62)	*p* value
*CFR (%)*			
2 cm above the LT tip (A1)	61.56 ± 6.22	62.15 ± 5.91	0.247
LT tip (L)	78.93 ± 8.27	79.75 ± 6.36	0.559
2 cm below the LT tip (B1)	82.89 ± 6.33	81.83 ± 6.19	0.297
7 cm below the LT tip (B2)	77.05 ± 9.74	71.02 ± 9.74	< 0.001
CFI	3.79 ± 0.71	3.95 ± 0.86	0.389
Preoperative ΔHCOR (mm)	1.19 ± 5.17	1.31 ± 5.09	0.884
Preoperative ΔVCOR (mm)	1.44 ± 3.55	1.32 ± 3.25	0.705
Preoperative ΔFO (mm)	− 2.71 ± 8.14	− 3.23 ± 9.02	0.923
Postoperative ΔHCOR (mm)	− 2.36 ± 3.92	− 2.06 ± 3.35	0.305
Postoperative ΔVCOR (mm)	2.11 ± 3.72	1.74 ± 3.24	0.455
Postoperative ΔFO (mm)	0.24 ± 7.45	− 0.46 ± 6.63	0.368
Preoperative LLD (mm)	− 5.52 ± 8.96	− 5.30 ± 7.70	0.947
Postoperative LLD (mm)	5.99 ± 7.36	7.01 ± 7.50	0.575

*CFR* canal fill ratio, *CFI* canal flare index, *LT* lesser trochanter, *HCOR* horizontal center of rotation, *VCOR* vertical center of rotation, *FO* femoral offset, *Δ* [affected side-contralateral side]

Multivariate logistic regression showed that high CFI (*p* = 0.014), low ΔVCOR (p = 0.012), and gender (*p* = 0.028) were independent risk factors for LLD 1 day postoperative, but postoperative ΔHCOR, ΔFO and CFR at any level had no effect on postoperative LLD (Table 3).

**Table 3:** Univariate and multivariate logistic regression analysis of postoperative radiographic LLD

	> 10 mm LLD	> 10 mm LLD	Univariate *p* value	Multivariate		
	− positive	− negative		Exp(*B*)	95% CI	*p* value
Age (year)	59.80 ± 9.08	61.23 ± 10.92	0.419			
BMI (kg/m^2^)	25.77 ± 3.49	25.52 ± 3.22	0.661			
*Gender (number, %)*			0.013	0.44	0.21, 0.92	0.028
Male	25, 51.02	80, 71.43				
Female	24, 48.98	32, 28.57				
*Side (number, %)*			0.572			
Left hip	23, 46.94	58, 51.79				
Right hip	26, 53.06	54, 48.21				
*Stem*			0.272			
Fully coated	27, 55.10	72, 64.29				
Proximally coated	22, 44.90	40, 35.71				
*CFR (%)*						
2 cm above the LT tip (A1)	62.36 ± 5.72	61.54 ± 6.25	0.432			
LT tip(L)	79.71 ± 6.95	79.05 ± 7.87	0.613			
2 cm below the LT tip (B1)	81.77 ± 6.79	82.80 ± 6.05	0.338			
7 cm below the LT tip (B2)	74.14 ± 11.40	74.99 ± 9.59	0.623			
CFI	4.08 ± 0.99	3.75 ± 0.63	0.014	1.77	1.12, 2.80	0.014
Postoperative ΔHCOR (mm)	− 2.50 ± 3.39	− 2.13 ± 3.85	0.566			
Postoperative ΔVCOR (mm)	0.76 ± 3.07	2.50 ± 3.61	0.006	0.86	0.76, 0.97	0.012
Postoperative ΔFO (mm)	0.63 ± 8.10	− 0.32 ± 6.68	0.437			

### Postoperative subjectively perceived LLD

There was no significant difference in the percentage of patients who subjectively perceived LLD between the two groups (Table 1). Multivariate logistic regression indicated that high CFI was an independent risk factor for postoperative subjectively perceived LLD (*p* = 0.013), while CFR and acetabular component positioning had no effect on subjectively perceived LLD (Table 4).

**Table 4:** Univariate and multivariate logistic regression analysis of subjectively perceived LLD after THA

	Subjectively perceived LLD-positive	Subjectively perceived LLD-negative	Univariate *p* value	Multivariate		
				Exp(*B*)	95% CI	*p* value
Age (year)	61.23 ± 9.84	60.63 ± 10.62	0.745			
BMI (kg/m^2^)	25.47 ± 3.80	25.65 ± 3.10	0.762			
*Gender (number, %)*			0.172	0.56	0.27, 1.19	0.132
Male	25, 56.82%	80, 68.38%				
Female	19, 43.18%	37, 31.62%				
*Side (number, %)*			0.450			
Left hip	20, 45.45%	61, 52.14%				
Right hip	24, 54.55%	56, 47.86%				
*Stem*			0.142	1.66	0.80, 3.46	0.177
Fully coated	23, 52.27%	76, 64.96%				
Proximally coated	21, 47.73%	41, 35.04%				
*CFR (%)*						
2 cm above the LT tip (A1)	61.54 ± 5.27	61.88 ± 6.39	0.754			
LT tip (L)	79.84 ± 6.91	79.03 ± 7.84	0.541			
2 cm below the LT tip (B1)	81.77 ± 6.00	82.75 ± 6.39	0.375			
7 cm below the LT tip (B2)	75.38 ± 11.52	74.49 ± 9.62	0.620			
CFI	4.12 ± 0.96	3.75 ± 0.66	0.008	1.82	1.14, 2.91	0.013
Postoperative ΔHCOR (mm)	− 1.69 ± 3.30	− 2.45 ± 3.84	0.246			
Postoperative ΔVCOR (mm)	1.45 ± 4.22	2.17 ± 3.24	0.252			
Postoperative ΔFO (mm)	0.38 ± 8.12	− 0.18 ± 6.75	0.658			

### Clinical outcomes

No statistical difference was found in terms of HHS 6 months (fully coated, 84.27 ± 7.70; proximally coated, 83.05 ± 7.28) and one year postoperative (fully coated, 93.39 ± 4.79; proximally coated, 93.21 ± 4.87) as well as FJS-12 6 months (fully coated, 63.59 ± 8.62; proximally coated, 62.90 ± 8.19) and 1 year postoperative (fully coated, 79.25 ± 4.79; proximally coated, 78.09 ± 4.74) between the two groups (Table 1). Multivariate linear regression showed that CFR at B1 (*p* = 0.017) was an independent risk factor for the HHS 6 months postoperative (Table 5). However, CFI and postoperative acetabular component positioning had no effect on HHS 6 months postoperative. No risk factor was found for HHS 1 year postoperative (Table 5).

**Table 5:** Univariate and multivariate linear regression analysis of postoperative Harris Hip Score

	Univariate	Multivariate		
	*p* value	*β*	95% CI	*p* value
*Harris Hip Score 6 months after THA*				
Age (year)	0.161	− 0.11	− 0.19, 0.03	0.172
BMI (kg/m^2^)	0.714			
Gender	0.349			
Side	0.576			
Stem (Proximally coated vs Fully coated)	0.318			
CFR (%)				
2 cm above the LT tip (A1)	0.155	0.15	− 4.22, 40.32	0.111
LT tip (L)	0.526			
2 cm below the LT tip (B1)	0.046	− 0.2	− 43.01, − 4.24	0.017
7 cm below the LT tip (B2)	0.923			
CFI	0.155	− 0.06	− 2.28, 1.09	0.486
Postoperative ΔHCOR (mm)	0.640			
Postoperative ΔVCOR (mm)	0.526			
Postoperative ΔFO (mm)	0.373			
*Harris Hip Score 1 year after THA*				
Age (year)	0.692			
BMI (kg/m^2^)	0.76			
Gender	0.113	0.09	− 0.76, 2.47	0.298
Side	0.254			
Stem (Proximally coated vs Fully coated)	0.814			
CFR (%)				
2 cm above the LT tip (A1)	0.34			
LT tip (L)	0.847			
2 cm below the LT tip (B1)	0.495			
7 cm below the LT tip (B2)	0.107	0.1	− 2.68, 12.40	0.205
CFI	0.652			
Postoperative ΔHCOR (mm)	0.763			
Postoperative ΔVCOR (mm)	0.095	0.12	− 0.05, 0.37	0.142
Postoperative ΔFO (mm)	0.789			

*CI* confidence interval

## Discussion

LLD is a common complication after cementless THA [9, 12, 32]. Patients with larger radiographic LLD and those with self-perceived LLD usually present with limping and more postoperative dissatisfaction [33]. However, the roles of proximal femoral morphology, femoral prosthesis filling, acetabular prosthesis positioning and stem design are not yet completely understood. In this study we found that high CFI, low ΔVCOR and gender were independent risk factors for postoperative LLD. Although CFR didn’t affect LLD, CFR at B2 had an effect on clinical outcomes. No statistical differences in early clinical outcomes or LLD were found between the two prostheses.

Proximal femoral morphology is important to help the surgeon to select the appropriate implant and to improve long-term survival [34]. However, an increase in postoperative LLD can have a negative impact on clinical outcomes [35], it has been shown that every 5-mm increase in LLD is associated with significantly worse clinical outcomes [11]. In our study, high CFI correlated with large postoperative LLD. Similar to our findings, Brumat et al. [12] founded higher CFI was an independent risk factor for larger postoperative LLD in cementless stems with metaphyseal fixation, but not in stems with diaphyseal fixation or cemented fixation. In contrast, Luger et al. [35] found that CFI did not affect the risk of increased LLD. The mechanism of CFI affecting LLD might be that a high CFI indicates a narrow femoral canal, which increases the resistance to driving the stem in, leading to an increase in the vertical protrusion distance and thus an increase in LLD. Besides the predictive effect of CFI on LLD, surgeons can select the appropriate prosthesis which can achieve a better medullary filling based on proximal femoral morphology before surgery. Several studies have shown that a better CFR can reduce complication rate such as prosthesis subsidence [1] and osseointegration failure [4]. Therefore, CFI remains the criteria for surgeons to select an appropriate prosthesis with a low complication rate.

It has been shown that leg lengthening after THA is related to the femoral stem positioning [36], which may correlate with femoral prosthesis filling. However, no study has demonstrated the existence of an effect of CFR on postoperative LLD. If CFR is found to have an influence on LLD, surgeons can select the appropriate prosthesis based on the role of CFR on LLD and thus improve clinical outcomes. Ishii et al. [4] found that narrow femoral canal, larger distal canal fill and smaller proximal canal fill were associated with poor radiographic outcomes that may lead to failure of osteointegration and proximal–distal mismatch. However, our findings showed that none of the levels of CFR was an independent risk factor for postoperative LLD. This is consistent with Luger et al. [35]. However, a better CFR can significantly reduce femoral prosthesis subsidence [1, 4, 19, 20], which may affect postoperative LLD [23]. Thus, although we did not find an influence of CFR on LLD in this study, CFR remains a criterion for surgeons to select an appropriate stem. Moreover, almost no studies have analyzed the correlation between CFR and postoperative LLD. We will continue to study their relevance in the longer term.

It has been shown that improper COR reconstruction may result in inconsistent leg length or even dislocation [37]. However, accurate reconstruction of the COR is difficult due to lack of clear anatomic landmarks intraoperatively and the COR will move inwards and upwards due to improper acetabular reaming which is to fit the acetabular prosthesis to the acetabulum [38]. In this study, a low ΔVCOR was the independent risk factor for a large LLD, but no effect of ΔHCOR on LLD was found. Hirakawa et al. [13] found that the higher the COR of the hip was placed, the lower the length of the affected limb was correspondingly, which is similar to our findings. The principle we speculate is explained as follows. Since the femoral stem and the femoral canal fit closely together, they can be considered to move as a whole. A lower ΔVCOR means that the femoral stem and the whole move distally relative to the contralateral side, thus increasing the distance from the lesser trochanter to the “inter-teardrop” line relative to the contralateral side and eventually lengthening the affected limb. A balanced or moderately increased FO compared to the contralateral side is a target for surgeons, as this can assist in increasing hip stability and reducing the risk of LLD or even dislocation [39]. However, no correlation was found between FO and LLD in this study.

To exclude confounding factors, we also analyzed the roles of demographic parameters. Regarding age, Carmona et al. [14] found that CFI decreased with increasing age, thus older people may present with smaller LLD. Al-Amiry et al. [15] found larger BMI caused lengthening of the affected limb. However, we found no correlation between age and BMI with postoperative LLD. Warnock et al. [16] found a higher percentage of leg lengthening in female patients after surgery, depending mainly on anatomical differences between men and women. The femur is smaller in female than in male, leading to a more conservative osteotomy of the femoral neck and an eventual increase in femoral height. Another study found that female had more anteverted and valgus hip [14], which may also cause women to have a larger LLD. In this study, we found that gender had a significant effect on postoperative LLD but was only applicable to fully coated stems. Postoperative LLD was significantly higher in women than in men (*p* = 0.015), thus women were susceptible to lengthening of the affected limb.

We considered that the reason why the significantly larger LLD in female was also related to the narrower femoral canal. Our study found that the width of the canal at A1 (*p* < 0.001), L (*p* < 0.001), B1 (*p* < 0.001) and B2 (*p* = 0.044) was significantly smaller in female than in male (Table 6). This may lead to an increased difficulty in driving the femoral prosthesis into the femoral canal, thus increasing the postoperative LLD. CFI did not have significant difference between female and male; therefore, we believed that gender did not influence LLD through CFI.

**Table 6:** Difference in width of femoral canal between male and female

	2 cm above the LT tip (A1)	LT tip (L)	2 cm below the LT tip (B1)	7 cm below the LT tip (B2)
Female (*n* = 56)	41.94 ± 5.60	25.65 ± 4.09	18.40 ± 2.99	14.21 ± 2.55
Male (*n* = 105)	45.12 ± 5.49	28.43 ± 4.81	20.50 ± 3.06	15.16 ± 2.68
*p* value	< 0.001	< 0.001	< 0.001	0.044

The same objective LLD may lead to different subjective perceptions among different patients [33]. O’Brien et al. [10] founded that gender had no effect on subjective perception of LLD. And we found that only higher CFI was an independent risk factor for postoperative subjectively perceived LLD. High CFI was a risk factor for both subjectively perceived LLD and radiographic LLD, and further study is necessary to identify the differences between their risk factors.

D'Ambrosio et al. [1] found that proximal femoral morphology and CFR at any level did not affect clinical scores. It has been shown that adequate reconstruction of the hip COR is critical for higher postoperative hip function and satisfaction, and accurate placement of acetabular components can significantly improve HHS [21]. Our results showed that proximal femoral morphology, acetabular prosthesis positioning, and all levels of CFR except for at B1 had no effect on postoperative HHS. Although correlation between CFI and clinical scores was not found in our study, CFI may ultimately influence clinical outcomes by affecting femoral prosthesis filling and subsequently osseointegration [1]. We also found that demographic characteristics and femoral prosthesis design had no effect on clinical outcomes.

Although coating distribution had significant difference in these two prostheses, there was no difference in CFR except for at B2, ΔHCOR, ΔVCOR and ΔFO between the two groups, thus the positioning of the two prostheses was almost consistent. Fully coated femoral stems obtain initial fixation in the cortical region of the diaphysis, nevertheless there may be a risk of proximal femoral atrophy and distal cortical hypertrophy [40]. The proximally coated stem achieves initial fixation at the metaphysis and secondary fixation at the proximal coating [4]. It has been shown that fully HA-coated stems can compensate for the lack of femoral broaching technique and successfully achieved osseointegration even with slightly loosened stems [41]. Brumat et al. [12] found that the use of cementless femoral stem with metaphyseal fixation increased the risk of postoperative LLD in patients possessing higher CFI. However, we found no effect of prosthesis design on the risk of increased LLD, or on other clinical outcomes (HHS, FJS-12, self-perception of LLD). And this may be related to insufficient follow-up time.

The present study has several limitations. First, this is a retrospective study containing a small sample, which may influence the reliability of the conclusion. Second, we used only CFI to describe the proximal femoral morphology, making the description of femoral morphology inadequate. Third, the use of 2-D radiographic parameter CFI to describe the 3-D proximal femoral morphology limited the description of proximal femoral morphology to the coronal plane. Although CT scans are more accurate [42, 43], CFI remains a relatively simple and convenient method commonly used in clinical setting for assessing proximal femoral morphology [12]. Finally, the follow-up period may be insufficient, and thus further study of risk factors for long-term clinical outcomes is necessary.

## Conclusion

This study showed that proximal femoral morphology and postoperative acetabular prosthesis positioning but not femoral prothesis filling affected the LLD. High CFI was an independent risk factor for postoperative LLD and subjectively perceived LLD, and low ΔVCOR was also an independent risk factor for postoperative LLD. Women were also susceptible to postoperative LLD. Longer follow-up should be performed to study the effect of CFR on long-term LLD. Orthopedic surgeons can predict LLD based on femoral morphology and select an appropriate prosthesis to reduce LLD.

## Data Availability

The final dataset will be available from the corresponding author.
